# Mycotoxins Biocontrol Methods for Healthier Crops and Stored Products

**DOI:** 10.3390/jof7050348

**Published:** 2021-04-29

**Authors:** Kristina Habschied, Vinko Krstanović, Zvonimir Zdunić, Jurislav Babić, Krešimir Mastanjević, Gabriella Kanižai Šarić

**Affiliations:** 1 Faculty of Food Technology, Josip Juraj Strossmayer University of Osijek, 31000 Osijek, Croatia; vkrstano@ptfos.hr (V.K.); jurislav.babic@ptfos.hr (J.B.); 2 Agricultural Institute Osijek, Južno predgrađe 17, 31000 Osijek, Croatia; zvonimir.zdunic@poljinos.hr; 3 Faculty of Agrobiotechnical Sciences Osijek, Josip Juraj Strossmayer University of Osijek, Vladimira Preloga 1, 31000 Osijek, Croatia; gabriella.kanizai@fazos.hr

**Keywords:** biocontrol, mycotoxins, *Fusarium*, *Aspergillus*, *Penicillium*, *Alternaria*, antifungal compounds, commodities

## Abstract

Contamination of crops with phytopathogenic genera such as *Fusarium*, *Aspergillus*, *Alternaria*, and *Penicillium* usually results in mycotoxins in the stored crops or the final products (bread, beer, etc.). To reduce the damage and suppress the fungal growth, it is common to add antifungal substances during growth in the field or storage. Many of these antifungal substances are also harmful to human health and the reduction of their concentration would be of immense importance to food safety. Many eminent researchers are seeking a way to reduce the use of synthetic antifungal compounds and to implement more eco-friendly and healthier bioweapons against fungal proliferation and mycotoxin synthesis. This paper aims to address the recent advances in the effectiveness of biological antifungal compounds application against the aforementioned fungal genera and their species to enhance the protection of ecological and environmental systems involved in crop growing (water, soil, air) and to reduce fungicide contamination of food derived from these commodities.

## 1. Introduction

Today’s agriculture relies on different agents to improve the health, yield, and nutritive value of crops. Small grain cereals (such as wheat, barley, oat, rye, and triticale) and maize are the main commodities grown all over the world in different climatic conditions. Areas affected by drought, humid areas, and high altitude areas can deliver favorable conditions to the population of pathogen fungi. Because of the wide spectrum of climatic conditions, cereals and maize can be contaminated with different pathogens resulting in mycotoxins. The application of chemicals may result in a reduction of fungal infection or mycotoxin contamination, but the sustainability of such application regarding ecological and environmental issues is not promising. Current trends question the safety of chemical agents used for the preservation of crops [1], as they are considered responsible for many carcinogenic and teratogenic toxic effects in humans and animals [2,3]. Natural and biological weapons applicable in the reduction of mycotoxigenic fungi and mycotoxins have been intensely investigated for many years. Not only the producers, but the consumers of cereal-based foods as well, are seeking natural ways to protect crops and to reduce the amount of fungicides in final products [4].

Global warming and climatic changes reshape the microbiome of cereals and maize in all corners of the world. Shifts in fungal species have already been reported by several authors across the globe [5,6,7,8,9]. Several *Fusarium* species are affected by rising temperatures, and not only in European countries. This became a serious marker for climatic changes follow-up and can be considered as an indicator of global warming. Shifts in fungal species and their adaptation to stressful conditions, such as drought and warmer temperatures, subsequently result in changes in secondary metabolites, mycotoxins, and plant defense metabolites that can be detected and quantified in small cereals and maize [10]. This challenges the possibilities of fungicide reduction. Namely, harsher environmental conditions intensify the production of different fungal and plant metabolites which calls for increased use of fungicide agents. However, the committed efforts of scholars are currently aimed toward the development of biological and natural agents that can be employed not only for the protection of crops from fungal infections, but to reduce the environmental damage to ecological systems where these crops are grown.

The aim of this paper is to give an overview of recent reports on the application of biological antifungal compounds against *Fusarium*, *Aspergillus*, *Alternaria*, and *Penicillium* fungal species which would enhance the protection, not only of the plant itself, but of ecological and environmental systems involved in crop growing (water, soil, air) as well.

## 2. Mycotoxinogenic Fungi and Affected Grains

The most familiar fungal species that are related to mycotoxin contamination of maize and cereals belong to genera *Fusarium*, *Aspergillus*, *Alternaria*, and *Penicillium* [11]. Table 1 shows most common commodities, fungi, and mycotoxins worldwide. A more detailed overview of fungal species and their mycotoxins is given in the following sections.

### 2.1. Fusarium Spp.

#### 2.1.1. Species Description

*Fusarium* spp. are designated as the most devastating species for small grain cereals, especially for wheat and barley, causing Fusarium head blight (FHB) [13,14,15,16,17,18,19,20]. Oats are generally less affected by *Fusarium* spp. than other cereals [21,22,23], but some regions (Scandinavia and Canada) encounter a serious problem with oat panicle blight [21,24]. Favorable conditions for head infections caused by *Fusarium* spp. include high humidity and temperatures above 20 °C [14,25,26,27]. According to Miller [28], *F. graminearum* is associated with wheat and maize grown in warmer areas, and *F. culmorum* with colder areas such as northwestern Europe, and the influence of temperature correlates with a prolonged period of warm weather with daytime temperatures above 30 °C. Even though several fungal species are related to head blight, *F. graminearum*, *F. culmorum*, and *F. avenaceum* are found to be dominant species in most parts of the world [19,27,29,30,31,32,33,34,35]. A significant increase in FHB caused by *F. poae* has been recorded for the last few years. It does not cause classical fusariosis-like symptoms (significant damage to kernel germination capacity), but still produces mycotoxins [34,36,37,38]. Other species can also be related to the pathogenesis of small cereals: *Fusarium sporotrichioides*, *Fusarium crookwellense*, *Fusarium roseum*, *Fusarium equiseti*, *Fusarium tricinctum*, *Fusarium oxysporum*, and *Fusarium langsethiae*, *Fusarium acuminatum*, *Fusarium fujikuroi*, and *Fusarium incarnatum* [23,27,39,40,41,42,43,44].

According to several sources [45,46,47], *Fusarium verticillioides* is a common fungal species that infects maize. The infection can occur via several routes. Often, the kernel gets infected through airborne conidia that can be found on the silks [48,49,50]. Usually, a small percentage of the infected kernels display symptoms of infection [51]. Another proposed infection pathway is systemically through the seed [52]. Systemic infection can start from fungal conidia or mycelia, inside the seeds, or on the seed surface. In this case, the fungus thrives inside the young plant, moves up from the roots to the stalk, and ends up in the cob and kernels. *F. verticillioides* is known to produce toxins that are potentially toxic to humans and animals. The most significant of these toxins produced by *F. verticillioides* are the fumonisins [46,49,53]. Fumonisins can be detected in symptomatic and asymptomatic maize kernels, and therefore the control of fumonisin contamination in maize has become a priority area in food safety research with distinct limits for maximum fumonisin levels in human food and animal feeds [54,55].

As reported by Oldenburg et al. [56], *Fusarium* species infecting European maize mostly belong to the sections Discolour and Liseola. Discolour prevails in colder and more humid areas and Liseola prefers a warmer and dryer climate. As in most grains, several *Fusarium* spp. can be detected on maize which can result in multi-contamination with mycotoxins.

#### 2.1.2. Disease and Mycotoxin Producton

*Fusarium* spp. can also affect maize with two diseases described as “red ear rot” or Gibberella ear rot (*F. graminearum*, *F. culmorum*, *F. avenaceum*, *F. cerealis*, *F. poae*, *F. equiseti*, and *F. sporotrichioides*), and “pink ear rot” or Fusarium ear rot, (*F. fujikuroi*) which takes place after pollination and is common in hot and dry climatic conditions [56,57,58,59].

#### 2.1.3. Gibberella Ear Rot

Gibberella ear rot starts at the ear tip after entry of the fungi through the silks at female flowering [60,61]. The infection results in a grey-brownish to pink-reddish coloration of the infected parts of the rachis. The coloration usually indicates places where mycotoxins accumulate. Earlier ear tissue infection results in higher mycotoxin concentrations. Higher mycotoxin concentrations can be found at the ear tip if the infection occurred via the silks [62]. According to Oldenburg and Ellner [62], harvested kernels placed at the tip segment of maize ears, if the inoculation with *F. culmorum* or *F. graminearum* occurred during the flowering period, can contain DON, 3-a-DON, and ZEN. In comparison, rachis parts showed several times higher levels of the same mycotoxins (DAS, T2, and HT2 can be detected less often and in much lower concentrations) [34,63,64].

#### 2.1.4. Fusarium Ear Rot

According to several sources [65,66,67], *F. temperatum*, can also be designated as a causative agent of ear rot in maize. Infection occurs more often through damaged tissue than through silks [68,69]. *F. verticillioides* causes tan to brown coloration, white or light pink mycelium on kernels, limited ear areas, or groups of kernels scattered over the ear [70]. Kernels can be infected with *F. verticillioides*, but show no visible symptoms of infection [62]. Common mycotoxins produced by *F. verticillioides* and *F. proliferatum* in maize ears are fumonisins (FB1 to FB4) [71,72,73]. FB1 synthesis in maize kernels correlates to the content of water, amylase, and starch [74]. FB1 accumulation in immature *F. verticillioides*-infected kernels was not observed due to the lack of starch [75]. Bluhm and Woloshuk [75] described amylopectin as a triggering substance to induce FB1 production. Higher FB1 concentrations were observed in kernels that suffered dual infection with *F. verticillioides* and *F. proliferatum* [76]. However, *F. verticillioides* produces significantly higher levels of FB1 than *F. proliferatum* [77]. Infections involving several other *Fusarium* species, *F. subglutinans*, *F. avenaceum*, or *F. equiseti*, commonly result in different concentrations of MON (moniliformin), BEA (beauvericin), ENNs (enniantins), and/or other mycotoxins [34,64,78,79,80,81,82].

*Fusarium* spp. also cause seedling diseases such as seed rot, root rot, or seedling blight of maize [83]. Common causes of seedling diseases are *F. verticillioides*, *F. proliferatum*, *F. subglutinans*, *F. graminearum*, *F. oxysporum*, and *F. temperatum* [84,85]. Low-quality seeds and seeds that withstood significant damage by insects or physical damage are especially susceptible to soil- and seed-borne pathogens. Seedling blight can be recognized by the brown coloration of the dead seedlings or by light-yellowish coloring and seeds that have lost the capacity to thrive [56].

As reported before, *F. graminearum* prefers warm and hot climatic conditions (T > 15 °C). However, it can proliferate in a milder climate with higher temperatures and high humidity. *F. graminearum* is currently reported as the most common causal agent of head blight in cereals and maize ear rot [13,14,15,16,17,18,19,20]. *Fusarium fujikuroi* also prefers a warmer climate with hot and dry vegetation seasons [86]. *F. avenaceum*, *F. culmorum*, and *F. poae* are seen in colder parts of the world [85,87,88,89,90] with an average annual air temperature between 5 °C and 15 °C and moderate precipitation. *F. culmorum*, however, is much more harmful to cereals at higher temperatures [15,24,87]. *Fusarium* spp. are the main reason for seedlings’ death, foot rot, and head blight. Fusarium head blight (FHB) is a dangerous infection due to the subsequent mycotoxins contamination.

Infection of cereal heads with *Fusarium* spp. can occur at different times, but they are most susceptible to infection during the flowering phase and immediately after flowering. Warm and humid weather, dew, and higher precipitation during this period [26,29,91,92] enable the infection. Symptoms of infection show off on the infected spikes; they become white. The infected spikelets die out and block the development of kernels, resulting in a smaller, gray, shriveled, and loose consistency, and sometimes grains are covered with sporodochia and *Fusarium* spp. mycelium grains [26,29,36]. Infected grains are usually reddish in color.

Deoxynivalenol (DON)-producing chemotypes of *F. graminearum* are widespread around the world, while nivalenol (NIV)-producing chemotypes can be found in Asia and Europe. However, the occurrence of individual chemotypes is often affected by weather conditions [30,93,94].

### 2.2. Aspergillus Spp.

#### 2.2.1. Species Description

The most infamous fungi belonging to genera *Aspergillus* are *Aspergillus flavus* and *Aspergillus parasiticus*. Even though *Aspergillus* spp. can be found in small grain cereals, they prevail in maize and cause damage especially during droughty and hot seasons [95,96]. The reported climatic changes predict an increase of this pathogen, more severe infections, and significantly higher mycotoxin levels in cereals and corn [96,97].

*Aspergillus* spp. are commonly referred to as the black fungi, and they are pathogenic for several crops. Their habitat varies from temperate climatic conditions to tropical and sub-temperate zones. They can be found in soil, where they decompose dead plant tissue [95]. *Aspergillus* spp. can infect and cause serious economic damage to grapes, onions, maize, and peanuts. On maize, they cause maize seedling blight and maize kernel rot.

When combined with different hosts, some symptomless endophytes can act as pathogens or as saprophytes but, in either state, they can become producers of mycotoxins. Symptomless *Aspergillus* spp. infections have been reported in the literature but information about their ability to produce mycotoxins and any associated pathology is scarce. Early publications designated *A. niger* as the main species that causes damage. According to current findings, identification of *Aspergillus* spp. was somewhat off and certain corrections have been made. For example, today we know that the nomenclature of *A. niger sensu stricto*, or, *A. niger* var. *niger*, was so far designated as *sensu lato* and usually refers to *A. niger*. There are more than 190 *Aspergillus* species that can be separated into several distinct morphospecies. Some of the separations were done according to their colors [96], but more accurate and precise separation via data sequencing resulted in eight subgenera [97], of which only *Circumdati*, the sections *Circumdati* (=*Aspergillus ochraceus* group), and *Nigri* (*A. niger* group) represent economically harmful subgeneras. *Aspergillus* in section *Nigri* have been taxonomically revised, which resulted with several new taxa, such as *A. niger* var. *niger*, *A. melleus*, *A. sulphureus*, *A. brasilensis*, *A. ostianus*, *A. petrakii*, *A. scletotium*, *A. carbonarius*, *A. aculeatus*, *A. japonicus*, *A. tubingensis*, *A. ibericus*, and *Eurotium herbariorum* [98,99,100], but none of them have been identified as responsible for any crop disease. The *Aspergillus* genus prefers the tropical belt and is even more frequent in subtropical to warm temperate zones [95]. They thrive in the forest and cultivated soils, and dislike desert soils. Nevertheless, *A. niger* var. *niger* can be found in forests, grasslands, wetlands, deserts, and cultivated soils [95]. As mentioned before, the rising global temperatures will greatly influence the population and shift the species within the *A. niger* group to the more northern geographical latitudes.

#### 2.2.2. Disease and Mycotoxin Production

Mycotoxins associated with *Aspergillus* subgenera or specific species pose a toxic threat to livestock, poultry, fish, and human health. Severe cases of poisoning, such as the Turkey-X disease of peanuts, caused by *A. flavus* and *A. parasiticus*, have been described in the literature [101]. The identification of aflatoxins as the toxicological agent [102] was the first step towards the solution of ensuring food safety. Another group of mycotoxins, ochratoxins, was also related to this genera. Today, they are reported as carcinogenic mycotoxins and are included in the legislation. Recently, ochratoxins have been reported in several other species of *Aspergillus* sections *Circumdati* (*A. ochraceus* group), and by *Eurotium herbariorum*, a member of the *Aspergillus* section (*A. glaucus* group) [103].

### 2.3. Alternaria Spp.

#### 2.3.1. Species Description

Genus *Alternaria*, in particular *Alternaria alternata*, is a frequent contaminant of different small cereals causing “black point” disease. Favorable conditions for *Alternaria* spp. include high humidity and frequent precipitation [76].

#### 2.3.2. Disease and Mycotoxin Production

A common symptom of this disease is the coloration of ears and grains with dark pigment, and melanin [104]. The black point mostly causes a decrease in milling quality of wheat, barley, and oats, and is not as significant as a yield reducer. However, the changes in flour and bran color have significant economic importance. Besides the discoloration, decreased nutritive value, and the loss of taste also significantly reduce the technological quality of cereal products [105].

*Alternaria triticina* can cause damages to ears and grains, but the disease can occur on leaves in the form of leaf blight lesions. *Alternaria* spp. are often reported as storage fungi, where they cause spoilage of small grains and small-grains-based products. Even though they enjoy humid (high water activity) and warm storage conditions, *Alternaria* spp. can proliferate worldwide, in both humid and semi-arid climatic areas. *Alternaria* spp. Have been reported on wheat, barley, oat, and rye [106,107,108,109]. In the Mediterranean countries, as well as in Estonia, Slowakia, and Argentina, the prevalent species are *A. alternata* and *Alternaria tenuissima*, reported on wheat [110,111,112,113], while *Alternaria infectoria* was reported in Norway [105]. *Alternaria triticina*, originally from Indian wheat, was also reported in Argentina [114,115]. Toth et al. [116] reported *Alternaria hungarica* as a novel species on Hungarian wheat, considering it a minor foliar pathogen with small economic importance. Serbia reported *A. alternata* and *Alternaria logipes* as wheat pathogens, while *A. alternata* and *A. tenuissima* were noted on spelt wheat [117,118].

*Alternaria* spp. produce mycotoxins with different toxicological properties. Since there were reports that certain *Alternaria* toxins could exhibit carcinogenic effects [119], the European Commission (EC) requested that the European Food Safety Authority (EFSA) provide a scientific opinion on the risks to animal and human health related to the presence of *Alternaria* toxins in food and feed [120]. Besides, *Alternaria* spores are considered to be one of the most prolific fungal allergens, and have been associated with respiratory allergies and skin infections [121,122,123].

According to different sources [124,125,126], *Alternaria* toxins can be sectioned into three main structural classes:

- dibenzo-α-pyrone derivatives: alternariol (AOH), alternariol monomethyl ether (AME), altenuen (ALT), altenuisol (AS);
- tetramic acid derivates: tenuazoic acid (TEA);
- perylene derivatives: altertoxins I, II, III (ATX-I,-II,-III).

So far, *Alternaria* toxins have been detected in small cereal grains and small-grains-based products (bread and rolls, muesli, fine bakery wares, pasta, etc.) [120]. AOH, AME, and TEA in “black point wheat” on the German market [127,128]; AOH, AME, and ALT in Slovakian [113] and Czech grains [129]; AOH and AME in small cereal grains in Poland were reported [109]. Li and Yoshizawa [130] found wheat kernels significantly infected with mostly *A. alternate*; AOH was detected in 20 of 22 tested samples between 116–731 μg/kg. AME was at a mean level of 443 μg/kg (range = 51–1426 μg/kg) in 21 samples. TEA, the most abundant *Alternaria* toxin, was detected with an average level of 2419 μg/kg and with a maximum quantity of 6432 μg/kg. The presence of *Alternaria* strains in Argentinean wheat also designated TEA as the most abundant toxin [131].

### 2.4. Penicillium Spp.

#### 2.4.1. Species Description

Several *Penicillium* spp. (*Penicillium citrinum*, *Penicillium expansum*) have been reported as foodborne contaminants, but *Penicillium verrucosum* is one of the most concerning species belonging to genera *Penicillium*. It is generally assumed that *P. verrucosum* is a common producer of OTA in temperate and cold climates [132]. Even though much research has been conducted on *Penicillium* spp. on cereals and maize [133,134,135,136,137,138], this fungal genera is not as popular as the other selected genera in this review.

#### 2.4.2. Disease and Mycotoxin Production

*Penicillium* spp. are commonly saprophytic microorganisms that invade plant tissue and soil debris. Penicillium ear rot on maize usually occurs on ears already damaged by birds or insects [139,140]. In silage, the most frequently isolated *Penicillium* spp. is *Penicillium roqueforti* [141,142,143,144]. Based on rDNA genes analysis and chemotaxonomic profiles, a recent finding confirmed *P. roqueforti* as three species, *P. roqueforti*, *Penicillium paneum*, and *Penicillium carneum* [145]. Subsequently, only *P. roqueforti* and *P. paneum* have been reported in silage [143,145]. Both species, however, produce ROC (roquefortine C) and *P. roqueforti* also produces PR-toxin (Penicillin Roquefort toxin) and MPA (mycophenolic acid), while *P. paneum* produces PAT (patulin) as well [145,146,147]. Silage microbiota includes *P. expansum*, which produced ROC and PAT, *P. crustosum* and *P. commune*, both producers of CPA (cyclopiazonic acid), and ROC [148]. PAT and ROC can cause toxicoses in livestock (ROC has been reported as a suspected causal agent in several cases of paralysis, abortion, and placental retention in cattle) [141,142,149,150]. As with any other fungi, *P. roqueforti* can produce several mycotoxins at once, which makes it difficult to confirm that solely ROC is the toxin responsible for the reported symptoms. Several types of research suggested that ROC caused toxicosis in dogs after they had ingested food colonized by *P. roqueforti.* Reportedly, they suffered from paralysis, tremors, and convulsions [151,152,153], which indicates a neurotoxic effect. Here, too, it is impossible to claim that ROC is the main cause since another toxin, penitrem A, was detected as well. PAT was involved in cattle health disorders, causing tremors, paralysis, and death [154]. However, in this case, PAT was synthesized by *Aspergillus clavatus*, not *Penicillium* spp. Cattle suffered extensive damage to the nervous system. MPA is recognized as a potent immunosuppressant but does not possess the properties of acutely toxic compounds [155]. It is commonly utilized as an immunosuppressive agent for patients in need [156]. CPA, on another hand, is not well investigated but, in poultry, CPA exposure can result in tremors, liver, kidney, and gastrointestinal tract damage [157]. It can be excreted in milk, withstands pasteurization temperatures, and remains stable for extended periods of storage [158]. The potential dangers of *Penicillium* mycotoxins in the feed are yet to be fully discovered since not much information regarding their toxicity is available. CPA, MPA, PAT, and ROC are the most familiar toxins originating from *Penicillum* species (*P. roqueforti*, *P. paneum*, *P. commune*, *P. crustosum*, and *P. expansum*) [159].

Toxins are generally produced during storage time (low water activity, low pH, and oxygen concentration) [145,160,161]. For example, *P. roqueforti* and *P. paneum* can be found in silage. There they can thrive even if the silage is not visibly covered in mycelium, and they can also produce mycotoxins which is why they pose such a threat to animal and human health [141,145].

## 3. Bio-Acceptable Solutions for Fungal Control and Detoxification of Mycotoxins

Over the years, much scientific research has delivered various solutions for the reduction of fungal contamination, in the field as well as in storage silos. Current trends of sustainable development and ecological protection of nature, wildlife, and crops purport the reduction of chemical fungicides and the utilization of new and biologically acceptable substances originating from nature or non-chemical methods that can be applied to reduce or suppress fungal growth. This sustainability subsequently transfuses to human health protection. Table 2 presents a short overview of popular BCA methods.

### 3.1. Microbiological Approach

Recently, the application of various microorganisms (bacteria, yeast, and fungi) in the biotransformation of mycotoxins in food and feed has found many forms [162,277,278,279,280]. The idea to reduce the toxicity of a certain compound by transforming it into less toxic products with the mere use of eco-friendly subjects is more than enough to unite scholars in search of solutions. Popular biocontrol agents (BCAs) that are known to reduce FHB, belong to genera *Bacillus* [281,282,283,284,285,286], *Pseudomonas* [285,287], and *Streptomyces* [288]. Species belonging to *Cryptococcus* also display reduction properties and antagonistic activity towards pathogens causing FHB [289,290]. Different methods of biocontrol can be applied: antibiosis, mycoparasitism, competition, and the induction of resistance in the host plant [291]. The antagonistic activity of wheat endophytes is currently a popular weapon against *F. graminearum* [292]. Plants have different defensive compounds with which they act against pathogens. They are mediated by phytohormones such as jasmonic acid (JA), which triggers defense responses against necrotrophic pathogens; and salicylic acid (SA) which is activated by biotrophic pathogens. Both defense pathways interact antagonistically in the resistance response. The role of phytohormones in interactions involving pathogens, biocontrol agents, and the host are yet to be explored [293].

- Bacteria—some bacteria are known to bind and detoxify mycotoxins from different foods and beverages [162]. *Flavobacterium aurantiacum* B-184 was successfully investigated for the degradation of AFs capable of irreversibly removing aflatoxin from solutions. AFB1 can be detoxified via *Enterococcus faecium* by binding to the peptidoglycans and polysaccharides in the cell wall of the bacterium [163]. Aerobic oxidation and partitioning of DON into C3 carbon carried out by *Devosia* species reduces contamination with this mycotoxin [164]. *Lactobacillus* (L.) *casei* and *Lactobacillus reuteri* are known to bind AFs in aqueous solutions and *Lactobacillus amylovorus* and *Lactobacillus rhamnosus* display a binding efficiency of 60% AFB1 [165]. *Lactobacillus fermentum* was shown to be a satisfactory binder (98%) of FB1 and of T-2 (84%) [166]. *Bacillus velezensis* RC 218 and *Streptomyces albidoflavus* RC 87B successfully reduced FHB up to 30%, its severity up to 25%, and DON accumulation up to 51% on durum wheat under field conditions [167]. Zeidan et al. investigated the use of *Burkholderia cepacia* in the biocontrol of mycotoxigenic fungi and the reduction of ochratoxin A biosynthesis by *Aspergillus carbonarius*. The results indicated that QBC03 culture supernatant acted inhibitory to the growth of *Aspergillus carbonarius*, *Fusarium culmorum*, and *P. verrucosum*. Synthesis of ochratoxin A by *A. carbonarius* was also reduced [168]. De Melo Nazareth et al. reported that *Lactobacillus plantarum* CECT 749 CFS showed a high antifungal effect against *A. flavus* and *F. verticillioides* on corn kernels and corn ears, and FB1 and AFB1 levels were significantly reduced [169]. *Clonostachys rosea* (IK726) was used as biological seed treatment of cereals against *Fusarium culmorum* [170] and the results showed that this could be applied as an alternative to chemical fungicides for the control of seedborne infections caused by *F. culmorum*. *Clonostachys rosea* strain ACM941 was also tested as an anti-*Fusarium* agent and the results indicated that strain ACM941 of *C. rosea* is a promising biocontrol agent against *F. graminearum* and may be used as a control measure in an integrated FHB management program [171].
- Yeast—yeasts reproduce with great speed and produce antimicrobial compounds which act beneficially in humans and animals. The most popular yeast, *Saccharomyces cerevisiae*, can significantly degrade DON and reduce the rate of lactate dehydrogenase (LDH) release in DON-stimulated cells [172], but it can also reduce the levels of AFB1 and OTA [173]. PAT can be reduced by *S. cerevisiae* via physical adsorption where the O-N/N-H protein and polysaccharide bonds of cell walls interact with PAT [174]. *Kluyveromyces marxianus* can be useful in binding AFB1, OTA, and ZEN (zearalenone). *Candida utilis* can be applied in mycotoxins binding as well [175]. *Yarrowia lipolytica*, too, is very effective in reducing OTA concentrations (cca 50%) [176]. Another yeast, *Rhodotorula mucilaginosa*, is known to degrade PAT to dexipitulic acid [177]. The application of *Lachancea thermotolerans* in the control of *Aspergillus parasiticus*, *P. verrucosum*, and *F. graminearum* and their mycotoxins was assessed by Zeidan et al. [178]. They reported that yeast colonies reduced *Fusarium* growth and the synthesis of DON. Inactivated yeast cells were able to reduce almost 82% of OTA [178].
- Fungi—according to a source [163], fungi capable of producing aflatoxins can also break them down. Fungi such as *Aspergillus*, *Rhizopus*, *Trichoderma*, *Clonostachys*, and *Penicillium* spp. are proven to be successful in the detoxification of mycotoxins [179]. Non-toxic strains of *A. flavus* and *A. parasiticus* were shown to be very effective in reducing aflatoxin contamination in maize, cotton, pistachio, and peanuts, when released into the soil around the crops in large amounts. They compete with native soil toxic strains and prevail [180]. An extensive book chapter by [1] provides a more detailed insight in this topic.
- A commercially available combination of yeast, bacteria, and oomycete (*Trichoderma asperellum*, *Streptomyces griseoviridis*, *Pythium oligandrum*) was tested against *F. graminearum* and *F. verticillioides*. The results showed that against *F. graminearum*, *T. asperellum* was efficient in reducing the growth and mycotoxin concentration by 48% and 72%. 78% and 72% was the efficiency against *F. verticillioides*. *P. oligandrum* reduced the growth of *F. graminearum* and mycotoxin concentration by 79% and 93%. *F. verticillioides* growth and mycotoxin concentration was too reduced (49% and 56%). The application of *S. griseoviridis* resulted in a growth inhibition zone where the pathogen mycelium structure appeared to be altered, suggesting the diffusion of antimicrobial compounds [181].

### 3.2. Preharvest Agronomical Strategies

- Crop rotation—crop residues are an excellent habitat for fungi due to containing many nutritious residues. Without crop rotation, fungi reside on residues of previous crops and can transfer to the next commodity sown in the field. Crop rotation can help in the reduction of *Fusarium* spp. development and subsequently reduce the mycotoxins levels in grains [59,182]. Planting cereals year after year on the same field, especially after wheat and maize, facilitates the development and proliferation of *Fusarium* spp. [14,183,184,185]. In fields where wheat was sown after maize, DON levels in grain were elevated [186], but ZEA was detected in 45% of the samples [185]. Forecrops that act limiting to *Fusarium* spp. are root crops and legume plants [184,187]. According to [186,188], soybean as a forecrop reduces the Fusarium head blight and DON levels in wheat. The lack of crop rotation in conventional cereal cultivation presumably leads to a higher infection rate than in organic farming [24]. Another way of reducing *Fusarium* spp. infections is the use of catch crops. A catch crop is a fast-growing crop grown between successive plantings of the main crop. The cultivation of white mustard reduced the occurrence of *Fusarium* spp. and acted positively on the health of the main plant [189]. The removal of previous crop residues can also act favorably to *Fusarium* spp. suppression.
- Tillage—one of the most important methods for FHB reduction. Soil cultivation by tillage means that the topsoil up to 30 cm would reverse, or shallow up to 20 cm. This affects the reduction of mycotoxins in grains as well [24]. Inverting the soil with a plow and covering the plant residues from the previous crop proved to be a very efficient method for DON reduction [190]. According to [191], deeper tillage shows better results in the fungal count.
- Fertilization—interestingly, the application of mineral fertilizers in the field could induce a higher infection rate of *Fusarium* spp. [192]. Namely, due to the excess nitrogen content in the soil, the frequency of grain infection with *Fusarium* fungi becomes higher. However, even though the type of fertilizer (urea, ammonium nitrate, or calcium nitrate) can affect the rate of grain infected with *Fusarium* spp., DON levels are not as affected [193]. A study reported different mycotoxins in winter wheat fertilized with a higher nitrogen dose, 200 kg N ha^−1^, in comparison to the wheat treated with 120 kg N ha^−1^. A significant statistical relationship between the concentration of mycotoxins and the amount of nitrogen fertilizer and wheat cultivar was confirmed as well [194].
- Seed and sowing—sowing high-quality seed is an important factor in the prevention of pathogenic fungi. Healthy, undamaged seeds with adequate viability and appropriate moisture are desirable seed material [40]. The sowing date can significantly affect crop yield. During the flowering period, the risk of *Fusarium* infection is higher, therefore winter cereals are less susceptible to *Fusarium* infection [195,196]. An early sowing date of maize, in a moderate climate, can act protectively against fungal infections [197]. According to [198], high maize grain contamination with mycotoxins occurred while high precipitation and lower temperatures prevailed during the flowering to the maize maturation period. Maize infection and the accumulation of mycotoxins (fumonisins) is especially expressed during drought periods [34] which could be easily resolved by implementing an irrigation system. This helps the plant to relieve the stress caused by the drought which subsequently reduces the infection rate of *F. verticillioides* and mycotoxin contamination. For small cereal, irrigation can actually contribute to the occurrence of FHB [199,200].
- Breeding and selection—various genetic pools of breeding programs in individual countries, and agronomic and environmental cultivation conditions provide different genetic material [201]. So far, genetic modification has resulted in varieties resistant, or showing partial resistance, to *Fusarium* spp. This has proven to be the most suitable method for the suppression of *Fusarium* infections [21,202,203,204]. Mechanisms developed by cereals to defend from *Fusarium* spp. involve five types: type I is the resistance to infection and type II is resistance to the spread of the pathogen in the head [205,206]. Type III is the so-called resistance to DON (or the ability to degrade it). Type IV describes the plants’ tolerance to infection and the presence of DON and other similar secondary metabolites [207], and type V refers to resistance to the accumulation and degradation of mycotoxins in grain by transforming them into non-toxic derivatives or by blocking the biosynthesis of toxic metabolites [208,209]. To achieve successful breeding and transgenesis, it is important to understand the fundamental molecular relations between the host-pathogen and plant defense systems [204]. Overexpression of the HvNEP-1 gene (an antifungal gene) in the endosperm causes barley to be less susceptible to FHB infection. This leads to lower mycotoxin levels in the grain [210]. Silencing of targeted genes is an important tool for *Fusarium* spp. control in cereals. RNA interference (RNAi) is a natural mechanism that regulates gene expression, but host-induced gene silencing (HIGS) is a transgenic technology used to silence fungal genes on plants during attempted infection with successful reduction of infection [211]. This method relies on the ability of the host plant to produce mobile, small interfering RNA molecules generated from long double-stranded RNA that are complementary to targeted fungal genes and act as effectors and regulators of plant response to pathogens. To achieve and induce gene silencing, these molecules are to be transferred from the plant to fungi [212]. Mycotoxins levels in cereals can be lowered by choosing a resistant cultivar, and by reduction of mycotoxin accumulation and biosynthesis. Phenolic compounds, peptides or carotenoids, and pro-oxidative molecules such as hydrogen peroxide can have a regulatory effect on mycotoxins synthesis [208].

### 3.3. Post-Harvest

- Microbiological agents—as an alternative to chemicals, and natural in origin, this firstly refers to antagonistic microorganisms. Interactions between cereal plants and microorganisms have been detected and defined as potentially beneficial, for they can enhance defense mechanisms in plants [213]. However, fungi have the ability to synthesize different secondary metabolites (antibiotics) that act antifungal, antibacterial, and have insecticidal characteristics, thus interfering with the growth and proliferation of other microorganisms [214]. Treating maize seeds with *Trichoderma harzianum* T22 [215] could suppress the growth of *F. verticillioides* and subsequent fumonisin accumulation.
- Physical methods—the most popular and relatively simple method is grain moisture adjustment. Namely, grain moisture should be adjusted shortly after harvest to ensure minimal microbial activity. Microbial activity, especially by field and storage fungi, can be expressed through damaged grains which can be a result of husking [216,217]. This can lead to increased mycotoxin concentrations in grains. Unit operations such as sorting, washing, and milling can be included in reducing the mycotoxins concentration in cereals and cereal-based products [220]. Non-invasive methods involving UV light illumination or opto-electronic sorting can be used for sorting. Some of the mycotoxins accumulated in the surface tissues of grains can be removed by cleaning, husking, and removing residues [219]. Therefore, high concentrations of mycotoxins can be found in damaged grains, fine material, and dust [218,241]. Cleaning the grains’ surface prevents colonization by *Fusarium* fungi and accumulation of their mycotoxins. As mentioned before, adequate humidity and seed storage temperature plays an important role in mycotoxin levels and fungal proliferation [221]. Different adsorbents (activated carbon, aluminosilicates, or polymers) have proven to be very effective in toxin absorption in vitro and in vivo studies [222]. A somewhat expensive but efficient method that controls the fungal growth and the production of mycotoxins is antioxidants and essential oils applied alongside the utilization of a controlled atmosphere in the storage room [241]. Ozone application to disinfect cereals, vegetables, and fruits, or to detoxify mycotoxins [223], is increasingly used due to its simple application, the fact that it leaves no undesirable residues [224], and it is successful in preventing the development of pathogenic fungi during storage [225]. Ozonation can efficiently reduce DON content in wheat grain [226]. The exposure time to ozone is an important factor that determines the rate of mycotoxin degradation in grains [223]. The use of ozone (O_3_) in the degradation of mycotoxins was reported in several papers [223,227,228,229,230,231]. It is successful in the degradation of AFB1 and AFG1. Ozonification conducted under optimum conditions can significantly contribute to DON (29–32%), and its modified form DON-3-glucoside (DON-3-Glc) (44%), reduction [227].
- Radiation—commonly described as ionizing radiation or non-ionizing radiation [232] that can reduce or eliminate pathogenic microorganisms. Radiation can be utilized in industrial conditions, which makes it rather applicable for larger and bulk commodities. It changes the molecular structure of food ingredients with a series of reactions [219]. A very important discovery was noted in irradiated distilled water and fruit juices of orange, pineapple, and tomato contaminated with ZEN. Namely, ZEN toxicity was reduced. However, a higher dose of radiation (>10 kGy) affected the quality of the fruit juices [233]. Irradiation of 50 kGy with an electron beam caused degradation of ZEN and OTA by 71.1% and 67.9%, respectively, in naturally infected corn [234]. Gamma irradiation can also be applied and a reduction of AFB1 (>95%) at 6 kGy was recorded in rice processing [235]. PAT concentrations in apple juice were reduced by 83% after a 5 min irradiation [236]. However, the broader application of radiation methods in the food industry is still a questionable approach since it can cause physical, chemical, and biological effects following molecular reactions [234] that are potentially harmful to humans and animals.

### 3.4. Innovative Biocontrol and Detoxification Strategies

Nanoparticles—to reduce the toxicity of AFB1 magnetic carbon, nanocomposites have proven to be efficient, and nanoparticles of chitosan-coated Fe_3_O_4_ were utilized for PAT detoxification of PAT. Silver nanoparticles showed limiting properties against *Fusarium* spp. growth and have even proven to be effective in reducing mycotoxin levels [237,238]. A nanocomposites mixture of activated carbon, bentonite, and aluminum oxide showed excellent detoxifying properties for mycotoxins [239]. Research involving nanoparticles is on the rise and a new photocatalyst nanoparticle UCNP@TiO_2_ (upconversion nanoparticle) was reported to have the ability to degrade DON molecules in cereals [240].

Plant Extracts—essential oils (EOs) are composed of many bioactive compounds that can be applied as antifungal agents [242,243,244], but they can also be utilized to inhibit mycotoxin synthesis [227]. The application of natural agents is considered to be both human and environmentally friendly. Spanish paprika has an inhibitory effect on *A. parasiticus* and *P. nordicum*, and even on the production of AFB1, AFG1, and OTA. According to the source, the addition of 2–3% of Spanish paprika can help in reducing the AF and OTA in meat products [245]. An active paprika compound, capsaicin, reduced OTA production in grapes by *Aspergillus* section *Nigri* and by *A. carbonarius* [246]. Velluti et al. [247] studied cinnamon, clove, lemongrass, oregano, and palmarose essential oils on the growth of *F. proliferatum* and fumonisin B1 production in maize kernels. At 0.995 a_w_, all essential oils were effective at 20 and 30 °C. Lower a_w_ at 30 °C inhibited the activity of all essential oils while, at 20 °C, cinnamon, clove, and oregano oils were still active. FB1 production was inhibited by cinnamon, oregano, and palmarose oils at 0.995 a_w_ and both temperatures. Clove and lemongrass oils showed inhibitory activity at 30 °C. At lower a_w_, none of the essential oils were inhibitory to the production of FB1. Several scientists reported the inhibitory effect of clove and cinnamon oils on growth and aflatoxin production by *A. flavus* [248,249,250,251,252] and, in maize, cinnamon and clove oils effectively fought against aflatoxin formation by *A. flavus* for 10 days [250]. *A. flavus*, *A. ochraceus*, and *A. niger*’s reaction to oregano oil was noted by Paster et al. [253,254], and it was described as efficient in suppressing fungi in wheat [254]. Palmarose oil proved to be active against 12 fungi, to which it appeared to range from being non-effective to inhibitory [255]. Kanižai-Šarić [256] reported success in using different concentrations of butylated hydroxyanisole, propylparaben and thymol against *F. graminearum* on chicken and pig feed.

Cold plasma (CP)—is the so-called fourth state of matter mainly consisting of photons, ions, and free radicals (reactive oxygen and nitrogen species) with unique physical and chemical properties [257,258]. CP displays antimicrobial effects [219] and therefore it is commonly used in food processing [232]. Cold atmospheric pressure plasma (CAPP) is a low-cost and environmentally friendly technology that could be used for mycotoxin decontamination [219,258]. Low-pressure cold plasma was proven to be 50% efficient in the detoxification of alfatoxins on the surface of nuts [259]. Similarly, a significant reduction of AFB1 and FB1 mycotoxins was noted in maize to which CAPP was applied, and in a short amount of time (under 10 min) [258]. The application of cold atmospheric plasma resulted in a 93% reduction in AFs, 90% reduction in TCs, 100% reduction in ZEA, and 93% reduction in FUs after 8 min of exposure [257]. Plasma treatments in a duration of 5 s were reported to result in 100% degradation of AFB1, DON, and NIV [260].

Application of chitosan—a linear polysaccharide with antimicrobial characteristics in combination with a_w_ in fungal growth control and mycotoxin reduction by the *Fusarium* species (*F. proliferatum*, *F. graminearum*, and *F. verticillioides*) on maize and wheat was studied. The results showed a decline of DON and FB levels in irradiated maize and wheat grains following the application of low-molecular-weight chitosan with deacetylation above 70%, and a dose of 0.5 mg/g [261].

Marine microorganisms—a marine strain of *Pseudomonas aeruginosa* with good antifungal activity against *A. niger*, *A. flavus*, *A. oryzae*, *F. oxysporum*, and *Sclerotium rolfsii* was investigated by [262]. It was later discovered that it produces two broad-spectrum antifungal compounds (pyocyanin and phenazine-1-carboxylic acid) [263] that can reduce the growth of the above-mentioned fungal species. Chitinolytic marine bacterial strains, *Pseudomonas* sp., *Pantoea dispersa*, and *Enterobacter amnigenu*, showed characteristics applicable as antifungal biocontrol agents for the control of fungal plant pathogens, such as *Macrophomina phaseolina* and *Fusarium* spp. Gohel et al. [264]. Another chitinolytic microbial species isolated from water, *Streptomyces vinaceusdrappus*, displayed antifungal activity against sclerotia-producing pathogen *Rhizoctonia solani* [265]. A strain of marine *Bacillus megaterium* can effectively reduce the production of aflatoxins and expression of aflR and aflS genes [266]. In short, the analysis indicated that *A. flavus* genes were down-regulated by co-cultivation with *B. megaterium* across the entire fungal genome and especially within the aflatoxin pathway gene cluster (aflF, aflT, aflS, aflJ, aflL, and aflX). The expression of the regulatory gene aflS was down-regulated as well during co-cultivation and this resulted in the inability of the AflR/AflS-dependent aflatoxin pathway gene to transcribe and activate. In return, no AFs could be produced [267].

Marine yeasts—*Debaryomyces hansenii* was reported as an efficient species against pathogenic fungi. A strain of *D. hansenii* was able to reduce 80% of the incidence of disease caused by *Penicillium italicum* in Mexican lime [268]. *D. hansenii* was also studied against the mycelial growth of four maize postharvest pathogens (*Mucor circinelloides*, *Aspergillus* sp., *F. proliferatum* and *F. subglutinans*). The results were satisfactory since it was able to reduce the production of fumonisins of *F. subglutinans* to 59.8%, and postharvest decay by *P. citrinum* in Persian lime [269,270].

Fungi—some marine fungi and their secondary metabolites from marine fungi can be utilized as biocontrol weapons against pathogenic fungi. A cyclic lipopeptide (15G256γ) originating from the marine fungus *Hypoxylon oceanicum* was reportedly successful as an antifungal agent [271]. Numerous scholars [272,273,274,275,276] and their dedicated research are seeking biological compounds or microorganisms that could be applied in sustainable agriculture and food production.

## 4. Conclusions and Prospects

The future brings one inevitable thing—global warming. Climatic changes in combination with fungal shifts in cereals and maize will demand constant monitoring of food safety regarding different (regulated and so-far unregulated) secondary metabolites harmful to humans and animals. However, higher consciousness regarding environmental protection will probably delegate the ecological aspects of food production. Greater need for food and cereals will also influence the need to reduce ecological damage via fungicide application and will demand bio-acceptable solutions for bigger cropping areas.

Biological control is applicable and many novel methods are being discovered, mostly based on microbiological research and the application of microorganisms that can suppress fungal growth and detoxify mycotoxins. So far, agrotechnical measures have proven to be efficient if applied properly. Application of biocontrol agents should be done in storage units as well, rounding up the whole cycle from stable to table with good agricultural practice. Approachable methods are presented in this review, and for now the most reliable method is moisture reduction during grain storage. It is important to search for alternative methods and agents, to explore different approaches in ensuring food safety.

Bio-acceptable methods should be friendly to the environment and the crop, but also to producers and consumers. Introduction and application of biological and natural protective agents against fungal contamination will surely be one of the most important projects that would help ensure the health of humanity and help sustain eco-friendly food production.

## Figures and Tables

**Table 1:** Most common commodities, fungi, and mycotoxins worldwide.

Cereal	World Region	Mycotoxin	Fungi	Source
Wheat	Europe	Ochratoxin A	*A. ochraceus*, *A. carbonarius*, *A. niger*, *A. westerdijkiae*, *A. steynii*, *P. verrucosum*	[12]
	Central/South America, Europe, North Asia and South-Eastern Asia	Zearalenone	*F. graminearum*, *F. culmorum*, *F. crookwellense*	
	Europe and North Asia	T-2/HT-2 toxins	*F. sporotrichioides*, *F. langsethiae*, *F. poae*	
	Europe	Deoxynivalenol	*F. graminearum*, *F. culmorum*	
Rye	Europe and North Asia	T-2/HT-2 toxins	*F. sporotrichioides*, *F. langsethiae*, *F. poae*	
Barley	Europe	Ochratoxin A	*A. ochraceus*, *A. carbonarius*, *A. niger*, *A. westerdijkiae*, *A. steynii*, *P. verrucosum*	
	Europe and North Asia	T-2/HT-2 toxins	*F. sporotrichioides*, *F. langsethiae*, *F. poae*	
	Worldwide	Deoxynivalenol	*F. graminearum*, *F. culmorum*	
Oats	Europe and North Asia	T-2/HT-2 toxins	*F. sporotrichioides*, *F. langsethiae*, *F. poae*	
Maize	Common in Central/South America, Africa, South-East Asia; Occassional in North America, Europe and North Asia	Aflatoxins B1, B2, G1, G2	*A. flavus*, *A. parasiticus*	
	Europe	Ochratoxin A	*A. ochraceus*, *A. carbonarius*, *A. niger*, *A. westerdijkiae*, *A. steynii*, *P. verrucosum*	
	Central/South America, Europe, North Asia and South-Eastern Asia	Zearalenone	*F. graminearum*, *F. culmorum*, *F. crookwellense*	
	Europe and North Asia	T-2/HT-2 toxins	*F. sporotrichioides*, *F. langsethiae*, *F. poae*	
	Worldwide	Fumonisins B1, B2, B3	*F. verticillioides*, *F. proliferatum*	
	Worldwide	Deoxynivalenol	*F. graminearum*, *F. culmorum*	

**Table 2:** A short overview of biocontrol methods presented in this review.

Method				
Microbiological approach	Microorganisms	Bacteria [162,163,164,165,166,167,168,169,170,171]		
		Yeast [172,173,174,175,176,177,178]		
		Fungi [1,163,179,180]		
		Commercial agents [181]		
Preharvest agronomical strategies	Agro-technical measures	Crop rotation [182,183,184,185]	Forecrop [184,186,187,188]	
			Catch crop [189]	
		Tillage [190,191]		
		Fertilization [192,193,194]		
		Seed and sowing [195,196,197,198,199,200]		
		Breeding and selection [201,202,203,204,205,206,207,208,209,210,211,212]		
Post harvest			Microbiological agents	Fungus [213,214,215]
			Phisical methods	Moisture adjustement [216,217,218]
				UV light and opto-electronic sorting [219]
				Cleaning, husking and removing residues [220,221]
				Adsorbents [222]
				Ozonation [223,224,225,226,227,228,229,230,231]
				Radiation [232,233,234,235,236]
Innovative methods	Nanoparticles [237,238,239,240]			
	Essential oils [241,242,243,244,245,246,247,248,249,250,251,252,253,254,255,256]			
	Cold plasma [257,258,259,260]			
	Chitosan application [261]			
	Marine microorganisms [262,263,264,265,266,267]	Yeast [268,269,270]		
		Fungi [271,272,273,274,275,276]		

## Data Availability

Not applicable.
